# Practical Urinalysis with Clinical Hints

**Published:** 1887-03

**Authors:** 


### Practical Urinalysis with Clinical Hints. By J. B. S. King, M. D., Professor of Chemistry and Toxicology in Hahnemann Medical College, Chicago.

This is a series of eight charts or cards, nine inches by five and a-half, exposing a method of chemical examination of urine. They are excellently well arranged and are not too much abbreviated.

This method of putting the desired information in installments upon separate cards is an admirable one, and shows that the author is a practical chemist. A man who is manipulating chemical reagents, has his fingers moistened with them, and is probably holding test tubes and other utensils, objects to have to turn over the leaves of a book. But if he can have the desired text upon a card, as is done in the publication under consideration, he can, before beginning work, set up the cards in some convenient position upon his laboratory table, and then he need not disengage his hands from his work at all.

The writer of these remarks speaks feelingly, he speaks from experience, for he has "been there." We cordially recommend these cards. We suggest, though, that they should be sold packed in a stout manilla paper case to keep them together. W. M. J.

